# Edible Insects Consumption in Africa towards Environmental Health and Sustainable Food Systems: A Bibliometric Study

**DOI:** 10.3390/ijerph192214823

**Published:** 2022-11-11

**Authors:** Newton R. Matandirotya, Walter Leal Filho, Gaathier Mahed, Basil Maseko, Cleophas V. Murandu

**Affiliations:** 1 Department of Geosciences, Faculty of Science, Nelson Mandela University, Port Elizabeth 6000, South Africa; 2 Centre for Climate Change Adaptation & Resilience, Kgotso Development Trust, Beitbridge P.O. Box 5, Zimbabwe; 3 Research and Transfer Centre “Sustainable Development and Climate Change Management”, Hamburg University of Applied Sciences, Ulmenliet 20, D-21033 Hamburg, Germany; 4 Department of Natural Sciences, Manchester Metropolitan University, Chester Street, Manchester M1 5GD, UK; 5 Department of Food Science and Nutrition, Midlands State University, Gweru Private Bag 9055, Zimbabwe; 6 National Anglican Theological College of Zimbabwe, 11 Thornburg Avenue, Mount Pleasant, Harare Private Bag 2503, Zimbabwe

**Keywords:** edible insects, environmental health, sustainable food systems, food insecurity, malnutrition, bibliometric

## Abstract

Africa is home to an estimated wild edible insect population of 1000 species that offer an opportunity for sustainable food systems while also improving food and nutrition security on the continent. Edible insect consumption has been part of African communities for a long time and forms part of their diets and cuisines, particularly within low-income households with limited resources. The purpose of our study was to investigate and review the contribution that edible insects can make towards the realisation of sustainable food systems, and environmental/planetary health including the fulfilment of Sustainable Development Goal number 2 (zero hunger). Our study applied a bibliometric analysis approach using VOS Viewer, a data mining software. The study established that the consumption of edible insects is still widespread across many African countries and therefore can be used as an avenue for improving environmental health and enhancing food systems on the continent through a reduction in meat-based diets. This, in the long term, will also reduce the emission of greenhouse gases such as carbon dioxide and methane from livestock production-related activities. Edible insects are also known to contain a high percentage nutrient content of proteins, fats and iron and, thus, can also play a vital role in reducing food insecurity and malnutrition, particularly within low-income households. Due to the existence of a high number of edible insect species on the continent, communities in Africa can easily access sources that can further be preserved using various indigenous techniques while also having minimal impact on the environment. In addition, being a source of nutritious food, edible insects can also be a source of establishing sustainable livelihoods, as well as being able to be commercialised, thus further creating employment opportunities and economic growth. Some of the notable edible insects in abundance on the continent include termites, ants, crickets and caterpillars. Our study recommends that Africa should commercialise edible insect production, in addition to preservation processing that leads to the eradication of perennial food insecurity and malnutrition and improves environmental health, as well as developing sustainable food systems. We also further recommend the establishment of food safety guidelines on edible insects as most African countries do not have such a plan in place currently.

## 1. Introduction

Africa’s population is projected to grow to an estimated 2.4 billion people by 2050 [1], while the world population is estimated to reach 10 billion in this time [2]. This rapid population growth is going to trigger a rise in food demand by 60% [3] and, in some instances, food scarcity shortages. Currently, a considerable population is reliant on animal protein; however, reliance on animal protein is not sustainable due to its negative environmental impacts [4]. To compensate for the increase in population, there is a need to identify alternative sources of food to increase output; however, this requires more land and water that is not in abundance [5,6]. In addition to the resultant deforestation, soil erosion, water pollution and land degradation, other food production activities also emit considerable amounts of greenhouse gases (GHGs), such as methane and carbon dioxide [4], particularly livestock agriculture. It is estimated that 59–71% of global ammonia emissions come from livestock production activities [4], with estimates predicting a rise to 80% by 2050, with the main source being food production [7,8]. Climate change-induced stresses and shocks are also threatening food systems in Africa, for example, drought, floods and heat waves [9]. In addition to the climate change threats, unsustainable food systems in Africa are also causing destruction of the environment, which is particularly being driven by the need for large tracks of land for livestock production [4] and other animal-based protein.

In 2015, Sub-Saharan Africa contributed to a third of undernourished children globally [10], while 14 million were estimated to be experiencing conditions characterised by acute malnutrition [11]. Meanwhile, in the southern Africa region, Madagascar has been experiencing child-stunted growth and malnutrition, as one-third of men and women are anaemic [12]. This, therefore, shows that there is an urgent need to identify sustainable food systems with the capacity to produce sufficient food quantities to meet the needs of the growing population [7], while having minimal environmental destruction, for example, entomography. Entomography is the practice of edible insect consumption [13], and has been practised for years by some cultures worldwide as a method of providing unique, delicious and nutritious food to populations. Edible insect-based diets have the potential to create sustainable agricultural systems by reducing the nutrient and energy cycles, reducing biodiversity loss as well as reducing the rate of climate change [8]. To improve the environment, edible insects can be valuable in that they can convert solid waste into outputs that are useful, for example, organic matter [8].

On the other hand, edible insect diets offer a better alternative for food and nutrition security as they can be produced at lower environmental impact levels as compared to livestock [4], which requires more land–water resources [8]. Globally, it is estimated that there are 2500 insect species that are regarded as edible [14], and of these, Africa is home to an estimated 1000 species [15]. Insect consumption is also prevalent in Asia, Latin America, Africa and Australia, with 100 species reported as being consumed in Mexico alone (Lange and Nakamura, 2021) [8]. In Thailand, there are an estimated 20,000 edible insect farms [8], indicating a strong entomography culture. The reappearance of insects as a viable food group is not only attributed to their nutritional and economic value, but also to their environmental benefits [16]. Globally, it is estimated that two billion people depend on edible insect diets (entomography), especially during seasonal shortages [17]. Interestingly, there has been a notable increase in consumer interest in insect consumption worldwide [18], across both developed and developing countries.

Though entomophagy as a viable option to compensate for food shortages is being considered in both developing and developed countries [5], it remains underexploited. Entomophagy can contribute toward the realisation of sustainable food system goals [19], considering that most food systems across African countries remain vulnerable to various climate change-induced economic shocks and stresses [1,20]. Recently, the COVID-19 pandemic and the Ukraine-Russia conflict have exposed how vulnerable the African food systems are to external shocks.

Insect use as a food is mostly a common practice for people living in remote regions of developing countries, specifically in the tropical and sub-tropical climatic environments of Africa and Asia [21]. As a practice, entomophagy has hugely contributed to improving food security [22,23], as well as providing some safety nets for rural communities [24]. The study in Ref. [22] established that, due to high levels of vulnerability, rural communities consumed more edible insects than their urban counterparts. At a household level, edible insects are valuable mainly in two ways: they can be consumed by members or sold to earn extra income [25], therefore contributing toward economic empowerment and improved livelihoods [26]. Across Africa, the trading and street vending of edible insects is normally conducted by women, thus directly contributing towards economic wellbeing at the household level. Furthermore, insects have been regarded as beneficial to various communities in that they can produce silk, honey and wax, dyes and manna, are edible in nature, can be used as medicines and can be used to make beautiful ornaments [27].

**Figure 1:**
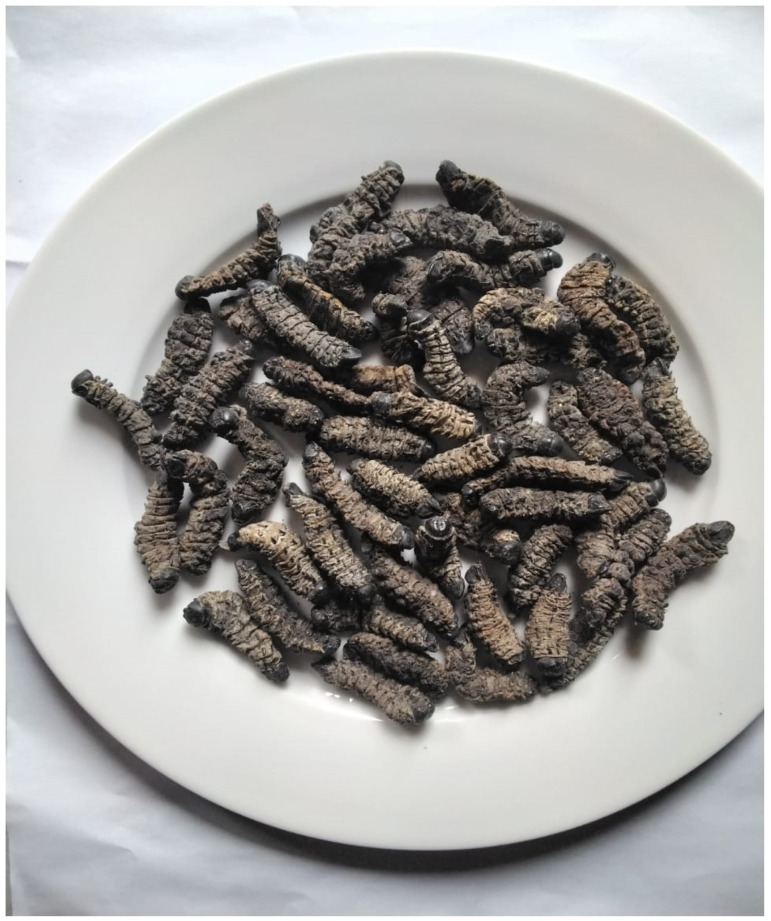
Mopane worms (*Gonimbrasia belina*).

Edible insects remain a sustainable source of income for low-income households across Africa [2,28], with most of the harvesting [29] and preparation for consumption being conducted by women [30], for example, the *Gonimbrasia belina (mopane worms) (*Figure 1). Across southern Africa, the mopane worms trade is estimated to be valued at USD 85 million [19], with the majority of trading being conducted by women. Part of the trading and livelihood creation involves street vending, which forms a key component of livelihood diversification [5].

The food and undernutrition adversity in Africa can directly be linked to diets that are widely unaffordable and staple foods with low nutritional content [31]. Undernutrition has devastating negative human health impacts, including reducing immune functionality and altering the gut microbiome [32,33]. Undernutrition during early life has been linked to chronic health challenges that include hypertension, diabetes and cardiovascular disease [34]. In addition, undernutrition in the form of micro-nutrient deficiency and protein-energy malnutrition are the most common types of nutrition insecurity in Africa. Nutrition interventions against these forms of undernutrition have focused on three main pillars, namely, food supplementation, food fortification and dietary diversity, as options to mitigate malnutrition [35]. Of the three, dietary diversification is the most sustainable, since it is environmentally friendly, and so becomes the most suitable option [35]. Dietary diversity involves the inclusion of various food types, some of which are underutilized/underexploited, as is the case with edible insects [5].

The purpose of our study was to investigate and review the contribution that edible insects can make towards the realisation of sustainable food systems and environmental health, including the fulfilment of Sustainable Development Goal 2 (zero hunger). The novelty of our study was to explore the potential linkages that edible insect-based diets can make towards setting up sustainable food systems in Africa, while also showcasing the research trends about edible insects, food systems and environmental health in Africa. Our study presents maps that highlight the interconnectedness of edible insects, food systems and environmental health across the continent of Africa, as well as the existing research gaps on the subject. Furthermore, our study presents an in-depth analysis on the nexus between edible insects, sustainable food systems and environmental/planetary health which, to the best knowledge of authors, has not been done before. Previous studies have mostly focused on entomography as a practice without identifying the various interlocutors on environmental/planetary health and sustainable food system benefits. Examples of past studies conducted in Africa include Ref. [23], which explored the utilization of indigenous knowledge in the consumption of a range of edible insects, while Ref. [2] investigated the contribution that edible insects can make towards sustainability. On the other hand, Ref. [36] explored the possibility of identifying edible insect mass producing approaches. Meanwhile, Ref. [14] investigated the various edible insects across the African continent.

Some of the countries where edible insects are popular include the Democratic Republic of Congo (DRC), Uganda, Nigeria, Malawi, Cameroon, South Africa, Botswana, Kenya, Rwanda, Namibia, Angola, Burkina Faso, Zambia and Zimbabwe [36]. Table 1 highlights some examples of edible insects across the African continent.

Table 1 highlights several edible insects that are found across different countries of Africa. It is evident that east, west and southern African communities already have extensive established edible insect consumption cultures. In some instances, the practice has been passed from generation to generation. The consumption of edible insects is most important as it assists in offsetting protein deficiency in most African countries, particularly within low-income households [48], as they are cheap and easily accessible.

Figure 1 shows mopane worms one of the edible insects that are mostly available in southern Africa. Mopane worms grow in *Colophospermum mopane* trees that are mostly found in the dry regions of southern Africa, including Angola, Botswana, Eswatini, Mozambique, Namibia, South Africa, Zambia and Zimbabwe.

**Figure 2:**
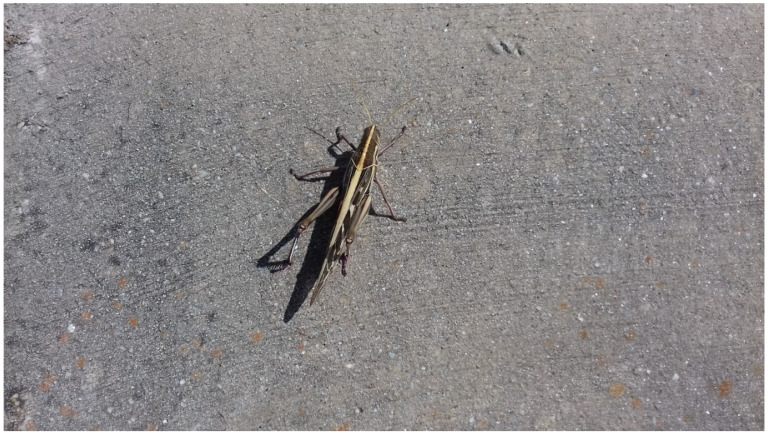
Grasshopper (*Eyprepocnemis plorans*).

Figure 2 shows a grasshopper, an edible insect commonly consumed in African countries, for example, Zimbabwe, Niger, Chad, Uganda, Sudan, Mali, Cameroon, Mozambique, Kenya, Malawi, the Democratic Republic of Congo, Zambia, South Africa, Burkina Faso and Botswana.

The grasshopper belongs to the family of terrestrial insects [49] that remain a very important source of food in Sub-Saharan Africa [49]. In a study in Ref. [50], it was established that 86.8% of respondents confirmed that grasshoppers were used as food, while 57.7% confirmed that grasshoppers were sold at markets for economic benefits. 

The culture around harvesting, preparation and consumption techniques has generally not been documented in most communities on the African continent; however, there is a strong existence of oral evidence [49]. In some instances, some communities have managed to maintain their edible insect practices (ranging from the way of harvesting, preparation and consumption), while on the other hand, some practices have been integrated due to migration and intermarriages between tribes, as well as modernisation. The oral transfer of knowledge on edible insects has, however, led to the loss of vital information over the decades, as none was documented.

In Africa, edible insects are consumed either as a staple, an emergency food source during times of food shortage, or an important delicacy [51]. The consumption of and preference for edible insects differ geographically [46,51]. For example, some people prefer consuming insects either fried, roasted or raw, and others may find eating insects not so palatable [51]. In Cameroon, one of the approaches used to harvest grasshoppers include shaking the dead leaves of the banana plant during the early times of the morning [49]. To prepare the grasshopper for consumption, the head and digestive parts are removed [49]. Furthermore, edible insects contain proteins, as their bodies are composed of 35% to 70% of dry matter [12], and most can be consumed after being boiled, roasted or fried, or even in their raw form [5]. Across cultures, edible insects are mostly consumed as a relish or as snacks after main meals. Meanwhile, roasting, cooking and frying are some of the common preparation approaches used for edible insects. In Togo, a majority of households prefer grasshoppers that are barbecued, while some households use preparation methods such as steaming, smoking, stewing and sometimes toasting [36]. Mopane worms are popular in southern African countries and are harvested at their larval stage [52], with the main harvesters being women who also take the role of preserving and cooking them at the household level. The most preferred approach for mopane worms is boiling and roasting [23].

## 2. Materials and Methods

This study used a bibliometric review approach, while VOS Viewer software (Centre for Science and Technology Studies, Leiden University, The Netherlands) was further used to analyse the data. Bibliometric analysis is grounded in the bibliometric theory to analyse literature through the application of mathematical and statistical quantitative methods [53]. The benefits that can be derived from bibliometric approaches are that they allow researchers to analyse a subject of interest through citations, co-citations and word frequency [54]. An internet article search was conducted within SCOPUS for published journal articles and chapters on edible insects in Africa, covering the period of 1995–2021. SCOPUS is one of the largest online databases [55] and is available at (https://www.scopus.com).

The study followed a three-stage procedure that included:

- data compilation;
- data arrangement and cleaning; and
- analysis, interpretation and visualisation [56].

The inclusion criteria for articles were that the article should have been published within the years of 1995–2021 and undergone a peer review process, while the exclusion criteria were that an article had not been peer-reviewed, or was a grey literature or report that had not undergone the peer review process or was an article published earlier than 1995 or later than 2021.

To select the appropriate articles, the study used a search string that had two anchors: “edible insects” and “sustainable food systems”. This was deployed with (“edible” OR “insects”* OR “Africa”) as the first anchor, while having all actors under sustainable food systems and environmental health as the second anchor (“sustainable” OR “food system” OR “environmental health” OR “Africa”). The search asterisk was used to optimise the search query for the articles with keywords that included edible insects, Africa and food systems, while quotation marks were applied to increase the accuracy of the search [55,57].

The study saved the article titles, year of publication, author’s names and nationalities and keywords as a TXT file in a comma-separated version format [53] from the SCOPUS database in Microsoft 2016. The initial search yielded a total of 302 articles, which were further manually screened [7] to exclude articles that were not related to edible insects or food systems and environmental health. The final analysis was performed on 156 articles within the VOS Viewer Software Version 1.6.18 environment (https://www.vosviewer.com), a free online text mining and bibliometric analysis software [58,59]. Within the VOS Viewer environment, the study applied ‘all keywords’ as the standard unit of analysis, while ‘full counting’ was used as the counting method [7]. The VOS Viewer software can be used to construct maps that show the co-occurrence and co-citation of authors [58].

## 3. Results

### Overview of Bibliometric Analysis

**Figure 3:**
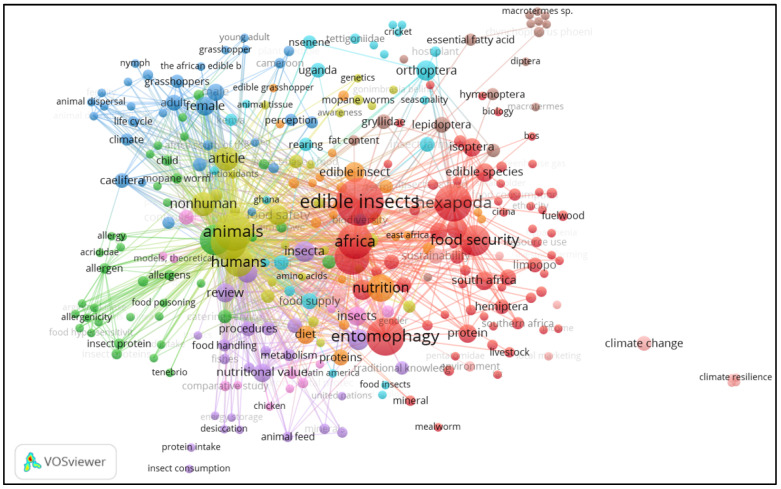
Output of keyword co-occurrence map of edible insect studies in Africa.

In Figure 3, keywords that featured prominently in the study are shown in three clusters: red, dark blue and light green. Within the three defined clusters, the keywords that were noted included edible insects, entomography, grasshoppers, beetle, termites and mopane worms. On the other hand, the countries where studies were conducted included South Africa, Uganda and Ghana. Close linkages were noted between such terms as Africa and edible insects, indicating that the studies focused on these two subjects.

**Figure 4:**
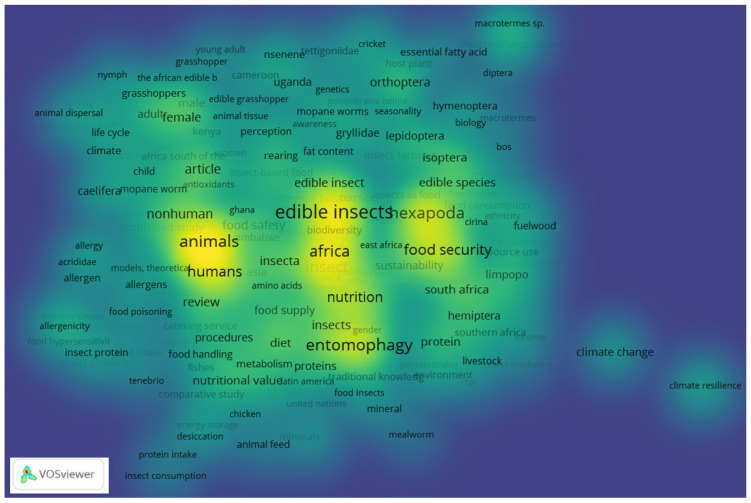
Density visualisation of key-word co-occurrence for edible insect studies in Africa.

The study established that entomography is widespread across the African continent, with different edible insect patterns. This was evidenced by the prominence of keywords that were highlighted in Figure 4 through the density visualisation map, wherein edible insects, Africa and entomography are shown in high-density colours. Mopane worms are quite a popular edible insect in the southern Africa region, particularly in Angola, Botswana, the DRC, Malawi, Mozambique, Namibia, Zimbabwe and Zambia [52]. The study in Ref. [23] established that 94% of participants in the province of Limpopo preferred mopane worms. In addition, being consumed at the household level, mopane worms also serve as a source of livelihood through trading [60].

**Figure 5:**
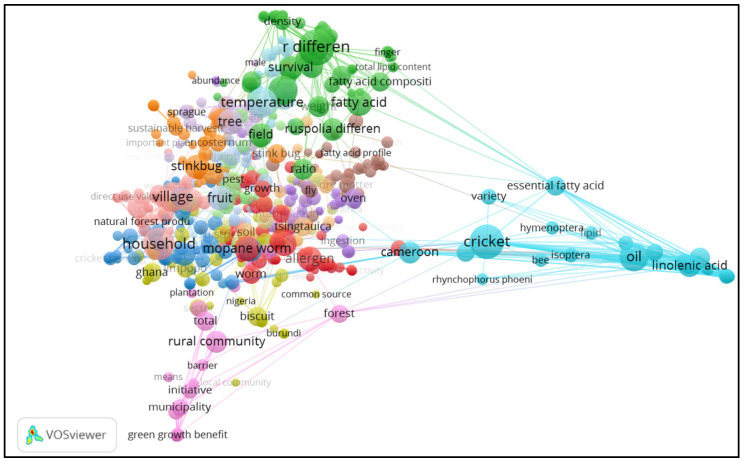
Output of term co-occurrence on edible insect studies in Africa.

Figure 5 shows the emergence of two main clusters that showed linkages between key terms from the studies, with close linkages being highlighted, indicated through a light blue cluster where the term ‘cricket’ is closely linked to ‘Cameroon’, ‘essential fatty acids’, ‘*Rhynchophorus phoeni*’, ‘allergy’, ‘alternative food source’ and ‘food allergy’. In the red cluster, the term ‘mopane worms’ is featured and linked closely to ‘tsingtauica’. On the other hand, the light green cluster indicates the prominence of ‘fatty acid’ and ‘fatty acid composition’, which are closely linked to ‘total lipid content’, ‘African edible bush cricket’, ‘edible grasshopper ruspolia’ and ‘edible ruspolia’.

**Figure 6:**
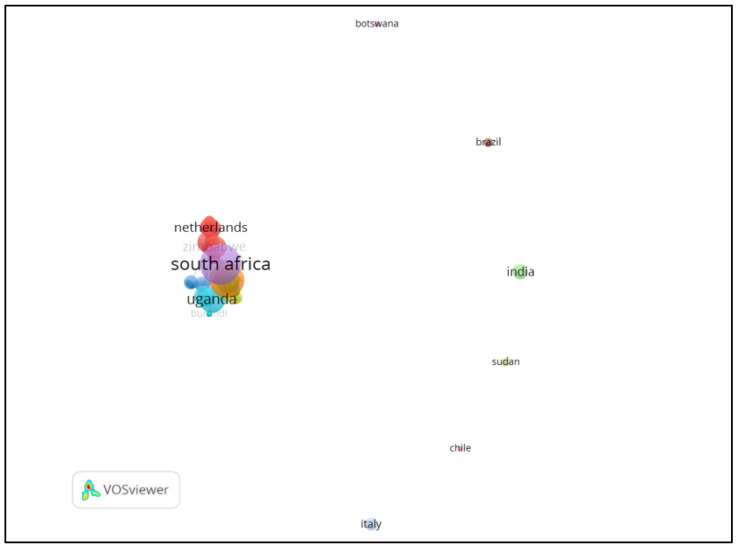
Output of country co-authorship on edible insects in Africa.

Figure 6 highlights the country-level co-authorship associations established by the study. The red cluster shows that co-authorships were mostly among researchers from Australia, Nigeria, Zimbabwe, the United States of America, Zambia and the Netherlands, while the yellow cluster shows co-authorships from Belgium, Burkina Faso, Canada and Congo. On the other hand, the purple cluster shows prominent co-authorships from South Africa, Namibia, Mali, China and the DRC. Co-authorship among researchers is a fundamental key to knowledge generation and development across the African continent.

## 4. Discussion

### 4.1. Environmental Health Benefits of Edible Insect Production and Diets 

Edible insects provide considerable environmental benefits, for example, by assisting in pollination activities, bio-degradation activities and humus formation, while also assisting in improving soil structure [27]. In addition, edible insects are also emerging as key in the recycling of organic waste into frass fertilisers that are of high quality in nature [61]. Some insects that have proven their capability in producing frass fertilisers include the black soldier fly, *P.sinuata*, *O.rhinoceros* and *H.illucens* [61]. The utilisation of insects in the recycling of organic waste promotes cleaner sustainable production systems and decreases environmental contamination [17]. Given these broad accruals from insects, edible insect farming can potentially curb the effects of climate change while conferring the environmental benefit of reduced greenhouse gas emissions [62].

Furthermore, edible insect-based diets present advantages including a low carbon footprint compared to beef, pork or poultry production [63] that results in a reduced need for land, a more rational use of energy and a reduced need for freshwater. According to [14,40], for a given 1kg of protein, edible insects require small pieces of land to be produced, compared to livestock production. Estimates indicate that 80% of GHGs emissions from agricultural activities are from livestock production, including methane (14%) from animal waste [14], the transportation of feed and clearance of land [64]. On the contrary, edible insect production produces lower air pollutant emissions, thus providing environmentally sustainable production pathways [64], as well as a low risk of zoonotic disease transmission to humans [43]. On the other hand, other mammals and bird species have a high capability to transfer disease to human beings [17].

The need for only small pieces of land means that insect farms can constitute an opportunity for livelihood diversification for low-income earning households [65], therefore reducing the level of environmental destruction and disturbances. Edible insect production is also attractive for its low cost of production [2,63], while being characterised by its only small environmental impact, and also allows for sustainable diets [14]. This is because insect farms require low initial capital outlay and equipment costs are minimal, therefore allowing for easy entry into the value chains [43]. The other environmental contribution of edible insects is that they can be used to improve sanitation while reducing pollution, for example, when flies’ larval and adult stages feed on the faecal waste matter [8]. The same mechanism can also be used to facilitate the recycling of livestock waste [63] by aiding the decomposition process. In addition, zoonotic disease transmission is quite limited between edible insects and humans as compared to between humans and birds or animals [17].

In addition, insects have single physiological as well as biological makeups that enable high feed conversion ratios (FCR) that enable the transformation of a given quantity of protein into animal protein [63,64]. Given the same quantity of feed between edible insects and other livestock, edible insects make the full efficient use of feed. It has also been established that insects have an exceptional ability to transform what they eat into viable tissues that can later be beneficial to other animal species [66], for example, insects have FCR that are twice as efficient compared to chickens and pigs, and five times more efficient than cattle [66]. The FCR of insects can also be further enhanced because they are poikilothermic (cold-blooded) in nature, unlike homeothermic (hot-blooded) animals [2,8,13,64], thus making them more environmentally friendly to produce.

### 4.2. Nutritional Contribution of Edible Insects 

Edible insects are high in both monounsaturated fatty acids and polyunsaturated fatty acids, coupled with an abundance of several minerals and vitamins [65] that are key in the provision of daily nutrient intake requirements to human beings. In some instances, the mineral and vitamin content of edible insects is higher in iron and zinc, compared to conventional meat [4]. Therefore, entomophagy has been proposed to combat the deficiencies of these minerals in developing countries [67], since 17% and 25% of the global population are at risk of zinc and iron deficiencies, respectively [68].

In Sub-Saharan Africa, edible insects with high consumption rates have been identified as beetles (31%), caterpillars (18%), bees, wasps and ants (14%) and grasshoppers, crickets and locusts (13%) [1,14]. Across the central Africa region, insects still provide more than 50% of dietary protein, and their commercial value is higher compared to animal-derived protein [69]. This can be attributed to the superior nutritional profile of numerous insects coupled with the ease of insect production and the low carbon footprint associated with insect rearing. As an alternative source of protein compared to domesticated animal-based foods, edible insects have additional benefits that include a high rate of reproduction [70] accompanied by superior nutritional content. Ref. [71] notes that edible insects have a wide range of nutrients that confer numerous health benefits to consumers. In the same spectrum, Ref. [71] regards edible insects as highly nutritious foods that can satisfy all human daily nutrient requirements. It is estimated that 67–98% of the protein contained in edible insects is highly digestible [5]. The protein content of dry matter in termites is estimated at 35%; crickets and grasshoppers contain 61%, while locusts contain 77% [17] and butterfly larvae containing 77.2% [72].

On the other hand, caterpillars have been noted to contain a high percentage of protein, fat, unsaturated fatty acids, mineral salts (iron, calcium and zinc) and various categories of vitamins [12,20,28]. A 100g sample of caterpillars was found to contain 76% of the daily nutrient intake requirements of adults [65]. During their larval phases, edible insects are believed to contain more protein content than red meat or fish [66], while at the same time also containing more fibre and omega-3 than beef [63]. A comparison with plant-based protein sources found that that edible insects also contain a higher protein content than soya beans [73].

Additionally, insects contain polyunsaturated fatty acids proportionate to fish and poultry; thus, they are a better, healthier choice for fats in the diet [66], as well as being high in fibre content [17] for a healthier livings. Ref. [65] reported high levels of phosphorus in edible insects that meet the adult’s dietary requirements. Besides grasshoppers contributing nutritionally, some communities in Chad and Sudan believe that they can cure many ailments such as high blood pressure, cancers of the stomach and jaundice [49], kidney disease and intestinal sickness [50], while in Zimbabwe, grasshopper powder is used to remedy bedwetting [49]. On the other hand, if they are not the main ingredient, grasshoppers when grided form an important traditional medicine ingredient [50].

### 4.3. Potential Contribution of Edible Insects to Sustainable Food Systems in Africa

It is estimated that approximately 500 species of insects are used as food in different countries in Africa [46], although the number could be as high as 1000; therefore, edible insects have the potential to significantly contribute to sustainable food systems across Africa. Edible insects remain a strong substitute for meat- and cereal-based diets, especially considering their high nutritional content and low cost of production. Recently, the COVID-19 pandemic almost rendered food systems non-functional during regional or national lockdowns that limited the movement of goods including food. This can be strengthened due to the already sizable population that maintains a tradition of edible insect consumption culture, as entomophagy is practised as a traditional heritage [68]. In the DRC, Ref. [74] reported that the Ngandu communities consumed 21 species of edible insects. The Bemba people of Zambia, southern DRC and north-eastern Zimbabwe were recorded to consume 30 edible insect species. In Kenya, insect species such as lake flies, ‘agoro’ termites, black ants, crickets and grasshoppers form part of the traditionally consumed meals in the western part of the country [37]. In some African countries, certain species are only consumed regionally, for example, stink bugs (*Hemiptera*: *Tessaratomidae*) are a delicacy for the VhaVenda people in the Limpopo province of South Africa [23]. The groups of edible insects consumed in South Africa are various *Lepidopteran* caterpillars, termites, grasshoppers, jewel beetles, ants and stink bugs [23]. In Kinshasa, DRC, it was estimated that the average household consumed approximately 300g of caterpillars per week, while 96 tonnes were consumed in the city annually as a major source of protein and other nutrients [75]. The already existing rich insect-eating culture can thus be exploited to strengthen the food systems of the African continent, while also ensuring cleaner production systems. This is the case because most of the harvest still takes place within the wild forest. Due to the need for less land, low capital outlay and low maintenance costs, edible insect farming can thus be expanded.

In the African context, edible insects foster livelihoods as they are sold in urban and rural markets, with a few favourites sold in urban markets [28], making them a vital source of income to rural communities; however, the absence of large-scale insect farms in Africa (except in South Africa) makes the collection of edible insects seasonal [4]. Therefore, the value of edible insects in many African countries fluctuates with the location, harvest season, type of insect and time spent searching for insects [1].

### 4.4. Toxicity and Food Safety Concerns Related to Edible Insect-Based Diets

Food safety is fundamentally key to human health, especially when dealing with new food sources. Food safety threats remain with the consumption of edible insects, for example, the insect itself could be toxic, the insect could have acquired pathogenic microorganisms during its life cycle, the insect could become spoiled after harvest and consumers can also experience allergic reactions to the insect [14]. The acquisition by edible insects of toxic substances or human pathogens is highly possible if not produced hygienically. Toxicity can also possibly be acquired through the bioaccumulation that occurs during pesticide usage in controlling pests [36], as well as other heavy metals. Pesticide residues therefore remain a threat to human safety [17], as wild insects can feed on sprayed crops [17]. Besides the bioaccumulation threat, some individuals are also allergic to certain edible insects [5,17].

Currently, most countries on the African continent do not have edible insect safety frameworks [14], including monitoring mechanisms [5]. Considering the host of microbiological and chemical hazards that come with insect diets, there is a need to encourage authorities to set these up, as most communities still rely on indigenous knowledge. Even though entomology has long been practiced on the African continent, food safety regarding edible insects remains a grey area, as not much is known about certain species [17] that are on the African continent. This remains a challenge as communities still lack adequate knowledge about which species can safely be harvested and consumed without risking poisoning. The lack of community awareness on which species can be harvested and consumed is further compounded by the lack of food safety guidelines that moderate the harvesting, preparation and trading of edible insects to avoid consumers being exposed to risk.

In South Africa, a general lack of national policy frameworks to blend in insects as food in a coordinated way is a gap that still exists [73]. Similarly, insects are not mentioned as food in the national regulations of most African countries [19], and there are few legal instruments to consider insects as food, even though insects have been consumed as food for decades in Africa.

The majority of edible insects still lack variety concerning their presentation, and dishes at home and on the market offer limited preparation choices; for example, most are presented as boiled or fried. In addition, when edible insects are poorly handled during preparation and processing, they emit an off odour that most people associate with bugs and hence can repel potential consumers. The off odour can be linked to possible rancidity, considering the high-fat content of insects.

Other issues that are associated with edible insect farming are the high cost of heating production sites, the high cost of pests and disease control and the high cost of hygiene maintenance [2]. In addition, insects that are introduced to be domesticated from their natural habitat are prone to infectious diseases that can be spread among host insect populations [76]. The disease-causing pathogens can easily be transmitted to human beings through workers in rearing facilities within the insect farms [76]. Some of the disease-causing pathogens include viruses, bacteria, fungi, nematodes and microsporidia [76]. The overharvesting of edible insects can also be an issue leading to a marked decrease in insect populations [40].

Across Africa, most harvests are being conducted from wild sources; therefore, some edible insects are facing extinction [17] from their over-collection without proportionate replacement. The overharvesting also negatively impacts other downstream insect activities such as pollination and humus formation. Furthermore, deforestation in Africa is causing the loss of insect habitat, thus threatening their population. Within African communities, termite habitats are also threatened by house construction activities, for example, brick moulding activities as sources of clay. In addition, the edible insect population is also being threatened by high environmental pollution from pesticides, insecticides and other heavy chemicals [25] that are used in both farming and industrial activities.

There are also cultural and social barriers to edible insect consumption [40], as some edible insects are not readily accepted due to some beliefs and myths held within communities. Furthermore, most of the edible insects are seasonal in nature, and when off-season, the prices are higher than other protein sources, for example, meat, thus making them unattractive [25]. The change in land use patterns in various African countries is posing some threats to mopane worms, for example, the conversion of land into agriculture, deforestation and decline in vegetation cover [77]. The use of wood as a fuel source threatens the survival of insects which are dependent on trees as their source of feed and habitat.

## 5. Policy Recommendations

Although, in some parts of Africa, there is still an insect phobia that remains a deterrent for potential consumers, it is fundamentally key that African countries increase educational awareness on the inherent nutritional value of edible insect consumption among communities. These awareness campaigns can be conducted in conjunction with the food industry, government food regulators, academia and NGO sector. The promotion of edible insects as an alternative source of food can go a long way in reducing food and nutrition insecurity across many African countries, while at the same time encouraging sustainable food systems. This is highly relevant when considering that the majority of edible insects are harvested from natural sources; therefore, if well supported, this could enhance the existing food systems that were recently challenged by the COVID-19 pandemic. Edible insect consumption can also act as a very reliable substitute for meat-based diets and can complement the cereal-based diets ever-threatened by climate change-induced shocks and extreme weather events. In addition, value addition pathways can also be developed that will further strengthen earnings at the household and small business levels. This value addition can thus include the correct labelling of edible insect food packages regarding the nutritional content in order to provide enough information to consumers and potential consumers.

It is apparent that, despite the existence of a very rich edible insect consumption culture in Africa, food safety and the risk of toxicity exposure also remain real threats to consumers, considering that most of the trading is conducted by informal traders who remain unregulated. Our study recommends the development of appropriate, country-specific edible insect guidelines that will work as a framework for harvesters, farmers, traders and consumers. The enactment of the edible insect guidelines can be accompanied with standardisation and quality control mechanisms that will form a policy framework within the edible insect sector. Since a majority of African countries still do not recognise edible insects as food, edible insects therefore remain underutilized and unexploited. To encourage edible insect growth in output, there is also a need to enact strong reforestation and afforestation policies that will enable the reestablishment of lost forest and vegetation cover. This will assist in the maintenance of edible insect populations, as some are currently threatened by loss of vegetation cover. African governments and the private sector should also put in place incentives and educational policies that encourage edible insect farming as a viable alternative to other forms of farming, considering the recognized aforementioned environmental/planetary benefits, including low greenhouse gas emissions.

## 6. Conclusions

This paper provides an overview on the extent to which edible insects are consumed in Africa, especially as a complement to diets. It emphasises the socio-economic aspects of using edible insects and describes the contribution that their use may provide towards handling food shortages. The practical implications of this paper are twofold. Firstly, it outlines the many advantages of pursuing the use of edible insects as a response to the current shortages seen in respect of conventional food sources, which are characterised by limited availability in rural areas and higher prices. Secondly, the paper shows that edible insect use offers a simple yet effective means to address some of the dietary needs of local populations, even though they cannot fully replace the dietary values of fresh fruits and vegetables.

The bibliometric analyses used analysed the available literature and identified some of the current trends, which illustrate the fact that the use of edible insects may increase in the future. The literature attests to the growing interest on the topic, and a variety of studies which have been trying to provide a better understanding on how various elements (e.g., the diversity of edible insects and their availability and cultural components) interact. Their use may also help to migrate some of the existing inequalities in Africa regarding access to food.

This paper has some limitations. The first is the fact that the bibliometric analysis only considered elements related to the use of edible insects and not of other alternative food sources. Secondly, the bibliometric analysis was not large enough to cover all types of edible insects being consumed in Africa today. Finally, the paper does not use detailed case studies which describe the impacts of using edible insects on the economy of African regions. The study also relied mostly on secondary published articles in one database of SCOPUS, leaving out other grey literature sources that were not published. This means that this study could have missed on some relevant studies. Furthermore, there were also inherent limitations in the use of bibliometrics, such as that although metrics distinguish between what is cited and what is not cited, these do not give credence to the quality of the literature.

Despite these constraints, this paper provides a welcome addition to the literature since it sheds some light on some of the issues which permeate the use of edible insects as food sources, and addresses the need to provide more information on their potential use in addressing the food needs of local populations. This could be an element which promotes action towards the increased use of edible insects, also as a response to changing climate conditions.

In respect of future trends, it is very important that adequate policies are put into place to assist communities to better take advantage of the use of edible insects. In addition, further studies which investigate how different methods could be deployed to harness the large-scale production of edible insects are needed, with a view to identifying appropriate measures to replicate their use across Africa.

## Figures and Tables

**Table 1:** Common edible insects of Africa.

Insect	Purpose	Community/Country	Reference
Black ant Smith *(Carebara* vidua)	Believed to hold medicinal and nutritional value. Mostly used by the elderly to treat various ailments	Luos, Kenya	[37]
Termites *(Macrotermes falciger, M.natalensis and M.michaelseni)*	Consumed as relish	Venda, South Africa	[38]
Winged termites	Medicinal purposes	Toro, Rwanda	[15]
Edible stinkbug (*Encosternum delegorgue Spinola)*	Consumed as relish	Zimbabwe, South Africa	[23,39]
Mopane Worms *(Gonimbrasia belina)*	Consumed as relish at the household level. Additionally sold as a source of livelihood	South Africa, Zimbabwe, Botswana, Angola, Namibia and Mozambique	[14,23,24]
Edible grasshopper (*Locustana and Zonocerous*)	Consumed as relish	South Africa	[23]
Wild silkworm (*Borocera cajani*)	Used for sericulture	Madagascar	[14,40]
African palm weevil (*Rhynchophorus phoenicis*)	Used as a delicacy	Congo Basin, Cameroon and South Nigeria	[14]
Cricket *(Gymnogryllus lucens)*	Eaten as a rich protein snack	Nigeria	[28]
Palm Weevil *(Oryctes monocerus)*	Eaten as a crunchy snack	Nigeria	[2]
Rhinoceros beetle *(Oryctes owariensis)*	Eaten as a main meal/snack	Congo	[41]
Winged Termite *(Pseudacanthotermes militaris)*	Traditional diet	Kenya	[2]
Longhorn Grasshoper *(Ruspolia Differens)*	Used to make soup	Uganda	[42]
Field cricket (Gryllus campestris)	Fried and grilled	Burkina Faso and Kenya	[43,44]
Crickets (*G.bima-culatus)*	Consumed as relish	Kenya and Uganda	[45]
Grasshopper (*Kraussaria angulifera*)	Fried and grilled	Burkina Faso	[44]
Soldier termites	Consumed after roasting	Benin, Cameroon, Kenya, Malawi, Tanzania, Zambia and Zimbabwe	[46]
Black ant (Cerebara vidua)		Kenya	[43]
Caterpillars (*Cirina butyrospermi*)	Cooked and consumed as relish	Burkina Faso, Uganda, Sudan, Guinea	[30]
Caterpillars (*Cirina forda*)	Cooked and served as snack or in vegetable soup	Ghana, Nigeria, Democratic Republic of Congo and South Africa	[47]
Bush cricket (*Ruspolia differens*)	Eaten raw, boiled, fried or sun dried and consumed as a delicacy	Kenya, Uganda, Rwanda, Tanzania and Madagascar	[47]
Scarab beetle (*Phyllophaga nebulosa*)	Cooked and served as a snack	Ghana	[31]

## Data Availability

Data will be made available from N.R.M. upon reasonable request.
